# Relationship between infarct gray zone and characteristics of ventricular tachycardia using multi-contrast delayed enhancement: preliminary results

**DOI:** 10.1186/1532-429X-11-S1-P50

**Published:** 2009-01-28

**Authors:** Gideon A Paul, Jay S Detsky, Kim A Connelly, Eugene Crystal, Alexander J Dick, Graham A Wright

**Affiliations:** 1 Sunnybrook Health Sciences Centre, Toronto, ON Canada; 2 University of Toronto, Toronto, ON Canada

**Keywords:** Ventricular Tachycardia, Implantable Cardioverter Defibrillator, Gray Zone, Delayed Enhancement, Implantable Cardioverter Defibrillator Implantation

## Introduction

Myocardial infarction (MI) consists of a central fibrous scar surrounded by a heterogeneous region of viable and non-viable myocytes. The peri-infarct gray zone provides the potential substrate for lethal ventricular arrhythmias via the reentry phenomena. The extent of the gray zone, characterised by delayed-enhancement MRI (DE-MRI), has been shown to be a strong predictor of mortality [1] and to the susceptibility for ventricular tachycardia (VT) [2] in patients with a history of MI. We have recently shown that quantification of the gray zone using conventional inversion recovery gradient echo (IR-GRE) images is sensitive to both image noise and the accuracy of the required manual contours of the blood pool [3]. A new multi-contrast delayed enhancement (MCDE) sequence [4] is an alternative approach to DE-MRI for which an automated data analysis procedure has been developed that provides more robust gray zone quantification [3].

## Purpose

In this work, preliminary results are presented examining the relationship between the extent of gray zone and inducibility of VT as well as VT cycle length.

## Methods and results

Eight patients (mean age = 67, all men) with an ischemic cardiomyopathy referred for implantable cardioverter defibrillator (ICD) implantation (according to MADIT II criteria [5] or for secondary prevention of VT) underwent a cardiac magnetic resonance (CMR) examination. Five of the eight patients had documented evidence of prior VT. All patients underwent assessment of cardiac function (cine SSFP), followed by viability imaging ten minutes after administration of 0.2 mmol/kg Gd-DTPA using both IR-GRE and MCDE sequences. The MCDE sequence uses a segmented SSFP acquisition following an inversion pulse, yielding 20 images at various effective inversion times. The IR-GRE gray zone analysis was performed using methods described by Schmidt et al [2] whilst for MCDE it was performed using an automated data clustering algorithm previously described [3].

**Figure 1:**
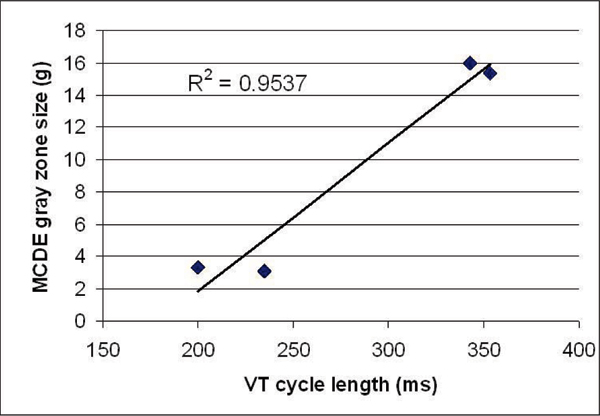
**Correlation between VT cycle length and MCDE-derived gray zone**.

VT was inducible at the time of ICD implantation in three patients (two of whom did not have any prior episodes of VT) and non-inducible in five patients. Imaging results within the two patient groups are shown in Table 1. There was a statistically significant difference in the size of the gray zone in inducible (16.3 ± 0.7 g) compared to non-inducible (6.6 ± 7.7 g) patients using MCDE (p = 0.046) which was not observed with IR-GRE (10.4 ± 4.6 g versus 7.0 ± 8.2 g, p = 0.48). There was no statistical difference between the two groups with regards to infarct core and total infarct size measured by either DE-MRI sequence or in the ejection fraction calculated using cine SSFP images. There was a strong correlation between the size of the gray zone as measured by MCDE and the VT cycle length (R^2^ = 0.95, Fig 1). Note that in one patient with a prior occurrence of VT, the cycle length is currently unknown.

**Table 1:** CMR-derived infarct characteristics for patients with inducible and non-inducible VT

		Inducible	Non-inducible	P
MCDE	Infarct core, g	18.3 ± 10.4	11.9 ± 14.8	0.50
MCDE	Gray zone, g	16.3 ± 0.7	6.6 ± 0.7	0.046
MCDE	Total, g	34.7 ± 11.0	18.5 ± 22.4	0.22
IR-GRE	Infarct core, g	17.3 ± 7.1	8.7 ± 10.9	0.22
IR-GRE	Gray zone, g	10.4 ± 4.6	7.0 ± 8.2	0.48
IR-GRE	Total, g	27.7 ± 11.7	15.7 ± 19.0	0.31
SSFP	EF (%)	25 ± 5	31 ± 19	0.55

## Conclusion

These results confirm that inducibility for VT is correlated to the size of the peri-infarct gray zone as assessed by MCDE. The higher variability in the IR-GRE analysis may contribute to the lack of a significant difference in the gray zone size between inducible and non-inducible patients in this small group of patients. We have also shown that the VT cycle length is correlated to the size of the MCDE-derived gray zone, consistent with previous results showing a positive correlation between VT cycle length and isthmus size measured during programmed electrical stimulation [6].
